# Predictors of Postoperative Bleeding After Cranial Surgery: The Role of Perioperative and Tumor-Related Factors

**DOI:** 10.3390/curroncol33010003

**Published:** 2025-12-19

**Authors:** Anatoli Pinchuk, Nikolay Tonchev, Anna Schaufler, Claudia A. Dumitru, Klaus-Peter Stein, Belal Neyazi, I. Erol Sandalcioglu, Ali Rashidi

**Affiliations:** Department of Neurosurgery, Otto-von-Guericke-University, 39120 Magdeburg, Germanynikolay.tonchev@med.ovgu.de (N.T.); anna.schaufler@med.ovgu.de (A.S.);

**Keywords:** hematoma, intracranial, postoperative hemorrhage, tumor surgery, blood loss, GOS, KPS

## Abstract

Postoperative hemorrhage (POH) is a rare but serious complication of cranial neurosurgery, often resulting in neurological deterioration and necessitating urgent surgical intervention. Despite its clinical relevance, POH remains underreported and insufficiently understood. This study aimed to identify potential risk factors including perioperative variables and tumor-related characteristics associated with POH requiring surgical evacuation. A total of 1862 cranial tumor procedures were performed in our department over a 10-year period. Data on perioperative parameters and tumor characteristics were retrospectively collected and analyzed. Statistical analyses were conducted to assess associations of them to POH. Statistical analysis revealed several peri- and postoperative variables significantly associated with POH in univariate analyses. These included intraoperative blood loss (*p* = 0.012) and length of postoperative hospital stay (*p* = 0.016). Furthermore, the outcomes measured using the Glasgow Outcome Scale (*p* < 0.001) and the Karnofsky Performance Scale (*p* < 0.001) showed also statistical relevance as a result of postoperative bleeding in these patients. The findings suggest that specific perioperative factors particularly intraoperative blood loss are associated with an increased risk of POH after intracranial tumor surgery. Additionally, prolonged hospitalization and worsened functional outcomes were linked to the occurrence of postoperative hemorrhage. In contrast, tumor-specific characteristics and routine laboratory values showed no significant association with hemorrhagic complications in this cohort.

## Simple Summary

Postoperative hemorrhage after cranial tumor surgery represents a serious complication with potentially profound effects on neurological function, recovery, and overall clinical outcome. To advance the understanding of clinical determinants and patient-specific characteristics that may increase the risk of such adverse events, we performed a retrospective evaluation of more than 1800 neuro-oncological patients treated in our department. Analysis of the collected data allowed us to identify clinically relevant associations to hemorrhage and tumor-associated factors that contribute to the development of this major surgical complication.

## 1. Introduction

Intracranial hematoma following craniotomy represents one of the most severe and life-threatening complications in neurosurgical practice. Its occurrence can result in elevated intracranial pressure, cerebral edema, local or global ischemia, and if untreated brain herniation [1]. Although factors such as intraoperative blood loss, inadequate hemostasis, and tumor pathology have been implicated [2,3], comprehensive data on the incidence and specific risk factors of POH after craniotomy remain limited. Most studies investigating intracerebral hemorrhage consider general patient populations, regardless of surgical intervention. As a result, there is a lack of focused research addressing risk factors for POH in patients undergoing craniotomy for tumor resection or hematoma evacuation. Furthermore, predictors of adverse postoperative outcomes in this specific subgroup remain insufficiently defined.

Medical and perioperative factors may also influence the risk of postoperative bleeding. Non-steroidal anti-inflammatory drugs (NSAIDs) such as ibuprofen and diclofenac are widely used for analgesia but carry a known risk of impaired platelet function, which contributes to POH [4,5]. Similarly, anticoagulation therapy, though often used for prophylaxis against venous thromboembolism (VTE), increases the risk of POH and remains a subject of ongoing clinical debate [6]. While the incidence of POH is lower than that of VTE, its consequences may be at least equal if not even more detrimental. Prophylactic anticoagulation is often recommended for patients undergoing brain tumor surgery, typically in combination with mechanical measures. However, the balance between preventing VTE and minimizing the risk of hemorrhage is delicate. Known risk factors for POH include a history of craniotomy, the use of heparin, and therapeutic anticoagulation [6,7,8]. Additional contributors include cancer-associated hypercoagulability and surgery-related factors such as endothelial injury, inflammation, and postoperative immobility [9]. Thromboembolic complications have been reported in up to 21% of patients within the first three months following craniotomy for brain tumors [6,9,10].

Numerous factors influence the postoperative clinical outcomes in patients with POH. Identifying the factors significantly associated with poor outcome provides a valuable reference for surgical decision-making, supports the clinical evaluation of postoperative prognoses, and may ultimately contribute to improved overall outcome in patients affected by POH.

Coagulation dynamics further influence hemorrhage risk. Any reduction in platelet count, whether pre-existing or iatrogenic, can increase the likelihood of postoperative hemorrhage [2]. Hemodilution due to the restoration of blood volume or blood transfusions can exacerbate thrombocytopenia and thus compromise hemostasis [11]. In a study by Adelmann et al., reduced postoperative fibrinogen levels were significantly associated with POH [12]. This aligns with findings by Wagner et al., which highlighted the impact of low platelet counts and coagulation factor deficiencies on hemorrhagic complications [13]. These mechanisms underscore the importance of perioperative coagulation assessment in neurosurgical patients.

Despite concerns regarding anticoagulation, some authors argue that POH is more often attributable to complication of surgery or underlying comorbidities rather than to chemical prophylaxis [14]. Wilhelmy et al. emphasized that although POH is clinically significant, thromboembolic events may exert a more detrimental effect on patient outcomes [14].

The risk of intracranial hemorrhage appears to increase with higher international normalized ratio (INR) values. For instance, when INR rises above 2.0, the likelihood of subdural hemorrhage increases markedly, whereas INR values between 1.0 and 2.0 are generally considered safe [15]. Wagner et al. further identified elevated INR as a predictor of hemorrhagic complications after cranial surgery [13]. Similarly, Fukumachi et al. found that partial tumor resection carried a higher risk of POH than complete excision [16], suggesting that the extent of resection is a relevant intraoperative factor.

Given the multifactorial nature of POH, accurate risk stratification is essential. Surgical trauma, pharmacological interventions, coagulation status, and tumor biology interact to influence postoperative outcomes. As background of this knowledge, we aimed to investigate the possible association between perioperative parameters and tumor characteristics with POH in patients undergoing craniotomy. Using univariate and multivariate analyses, our objective was to assess potential associations of hemorrhagic complications and functional outcomes, ultimately guiding individualized perioperative management and improving clinical decision-making.

## 2. Materials and Methods

A retrospective analysis was conducted on the medical records and radiological data of patients who underwent cranial tumor surgery at our institution between 2008 and 2018. During this period, 1862 patients underwent surgical resection of intracranial tumors. The review encompassed a wide range of clinical data, including tumor characteristics (tumor subtype, extent of resection, recurrence, and prior chemotherapy or radiotherapy), surgical parameters (operative duration, intraoperative blood loss, and transfusion requirements), and the incidence of POH and thromboembolic events.

Unless contraindicated, both preoperative and postoperative imaging was performed using contrast-enhanced magnetic resonance imaging (MRI). In cases of tumor-associated edema or significant mass effect, corticosteroids were administered preoperatively. Intraoperative adjuncts included ultrasound, electrophysiological monitoring, and, when indicated, frameless neuro-navigation.

Patients were excluded from the study if they were under the age of 18 or pregnant at the time of surgery. All postoperative imaging studies were meticulously reviewed for evidence of hemorrhage, which was the main outcome parameter in this study. Clinically significant postoperative hemorrhage was defined as bleeding that resulted in neurological deterioration, including increased intracranial pressure or mass effect necessitating surgical intervention. Associated symptoms included focal neurological deficits, headache, nausea or vomiting, and cognitive disturbances.

### Statistical Analysis

Categorical variables were reported as absolute frequencies and percentages, while continuous variables were presented as medians with interquartile ranges (IQRs), as all continuous data in this study were not normally distributed. Non-normality was confirmed using the Kolmogorov–Smirnov test. Associations between categorical variables and the occurrence of POH were evaluated using chi-square tests, or Fisher’s exact test when the expected frequency in any cell was below five. Differences in continuous variables between patients with and without POH were compared using the Wilcoxon–Mann–Whitney test. Variables were pre-selected based on a threshold of *p* < 0.25 in the univariable analysis and were subsequently entered as covariates into a multivariate logistic regression model. Potential multicollinearity among predictors was assessed using the variance inflation factor (VIF) and excluded if a VIF of 10 or higher was detected.

All statistical analyses were performed using SAS University Edition 9.4 (SAS Institute, Inc., Cary, NC, USA), SPSS for Windows, Version 18.0 (SPSS, Inc., Chicago, IL, USA), and R version 4.5.1 incorporating the packages gtsummary version 2.3.0 and stats version 4.5.1. Two-sided *p*-values less than or equal to 0.05 were considered statistically significant.

## 3. Results

**Figure 1:**
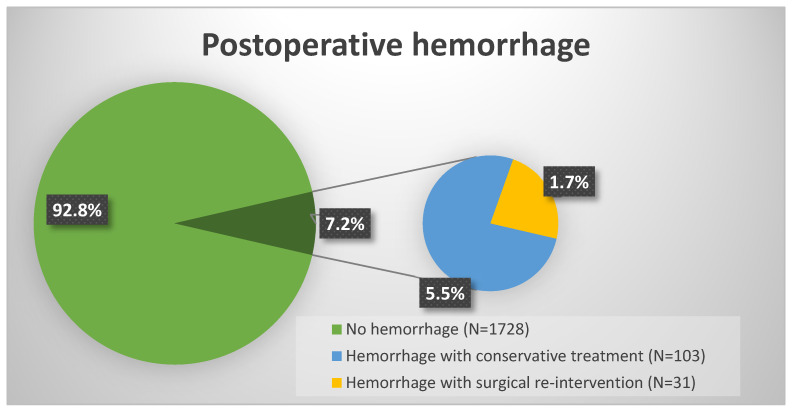
Postoperative hemorrhage following intracranial surgery: A total of 103 patients (7.2%) experienced POH. Of these, 31 patients (1.7%) developed hemorrhages severe enough to necessitate surgical re-intervention.

**Figure 2:**
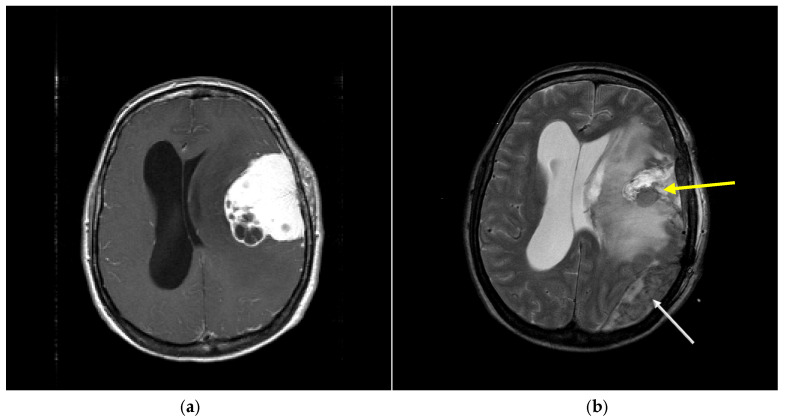
(**a**) Preoperative axial enhanced T1-weighted MRI revealed a large mass in the left parietal region. (**b**) Postoperative 24 h MRI showed total tumor resection with the tumor cavity hemorrhage (yellow arrow) as well as epidural hemorrhage at the left occipital area (white arrow). The presence of a second bleeding site makes surgical technique a less favorable risk factor for this hemorrhagic complication.

During the study period, a total of 1862 patients underwent intracranial tumor resection. Postoperative hemorrhage as primary outcome occurred in 134 patients (7.2%) (Figure 1). Surgical evacuation of the hemorrhage was required in 31 cases (1.7%). Figure 2 shows a POH example of individual hemorrhage localizations on postoperative cranial CT controls from this study.

### 3.1. Predictors of Hemorrhagic Complications

We investigated potential predictors of hemorrhagic complications in the entire study cohort. First, we assessed the effects of perioperative parameters, tumor characteristics, and preoperative use of NSAIDs, heparin and anticoagulation medications using univariate analysis.

### 3.2. Tumor Characteristics and Medications

Tumor characteristics included recurrence-operation (*p* = 0.637), tumor type (*p* = 0.972), tumor side (*p* = 0.749), tumor volume (*p* = 0.806), percentage of tumor resection (*p* = 0.079), radiotherapy (*p* = 0.833), chemotherapy (*p* = 0.888) were also evaluated. None of these parameters showed significant correlation. However, resection volume showed a trend towards significance, which can be explained with higher incidence of POH in patients with residual tumor in general. The more complex the tumor resection because of localization or tumor extension is, the more probable it becomes to experience such a postoperative complication.

**Figure 3:**
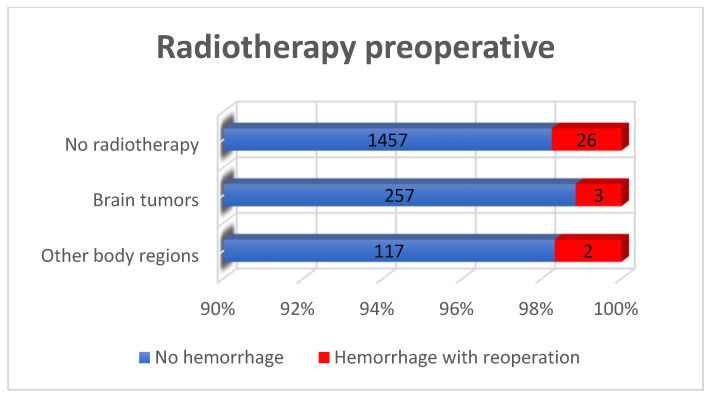
Postoperative major hemorrhage incidence in relation to preoperative radiotherapy: the history of radiotherapy treatment did not significantly influence the occurrence of postoperative hemorrhage among all patients (*p* = 0.833).

**Figure 4:**
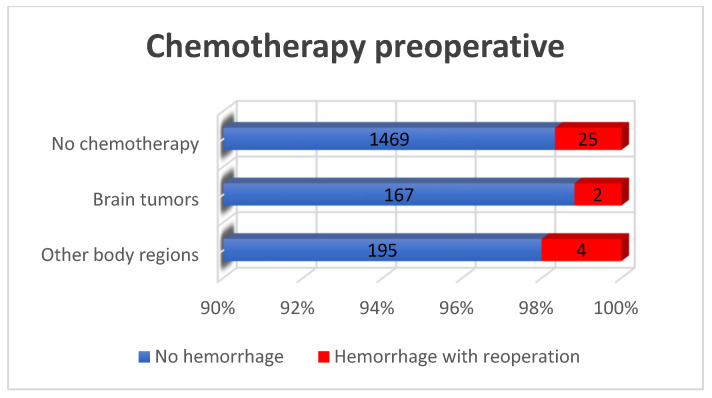
Postoperative major hemorrhage incidence in correlation to preoperative chemotherapy: The history of chemotherapy treatment did not significantly influence the occurrence of postoperative hemorrhage among all patients (*p* = 0.888).

The preoperative administration NSAID’s (*p* = 1.000), heparin in prophylactic doses (*p* = 0.224), and anticoagulant phenprocoumon (Warfarin^®^) (*p* = 0.326) did not demonstrate any statistically significant association with the occurrence of POH (Table 1, Figure 3 and Figure 4). These findings were probably influenced by integrated perioperative management protocols, which are standard for our institution.

### 3.3. Thromboembolism

A total of 34 patients developed postoperative thrombosis, and 46 patients experienced a pulmonary embolism following surgery. However, there was no statistically significant difference in the incidence of thrombosis (*p* = 0.438) or pulmonary embolism (*p* = 0.177) between patients with and without POH (Table 2).

### 3.4. Perioperative and Postoperative Factors

Several peri- and post-operative parameters were found to be significantly associated with POH. These include intraoperative blood loss (*p* = 0.012), length of postoperative hospital stay (*p* = 0.016), as well as outcome scores such as the Glasgow Outcome Scale (*p* < 0.001) and the postoperative Karnofsky Performance Scale (*p* < 0.001). In contrast, surgical duration (*p* = 0.111) did not reach statistical significance in this analysis (Table 3 and Table 4).

## 4. Discussion

Recently, Swedish study demonstrated a significant association between intraoperative blood loss and the development of postoperative hematoma [11,17], which was also supported by our findings regarding postoperative hemorrhage. Possible contributing factors include reduced levels of platelets and coagulation factors, which increase bleeding tendency, and excessive bleeding that leads to an overloaded surgical field, complicating the achievement of adequate hemostasis. Interestingly, another study showed that more extensive resection could reduce bleeding volume, as tumor tissue tends to be more vascular and prone to bleeding than normal brain parenchyma [18,19]. To minimize intraoperative blood loss and reduce postoperative hemorrhage risk, our institution implemented standardized perioperative hemostatic techniques and coagulation management protocols.

Mechanical hemostasis was achieved through tamponade with small gauze wads for localized bleeding control. Thermal hemostasis utilizing electrocautery was employed for vessel coagulation and hemostatic control. Local hemostatic agents, including gelatin sponges, fibrin glue, and other active flowable hemostatic substances, were applied as adjunctive measures to achieve optimal intraoperative hemostasis. Coagulation status was continuously monitored throughout the surgical procedure to identify potential coagulopathy. When coagulation disturbances were detected, targeted pharmacological therapy with coagulation factors and procoagulant medications was administered to support and optimize hemostasis. The tumor-associated characteristics, including tumor size, resection extent, and other perioperative and preoperative diseases, were not significant in our cohort and showed no relation to postoperative bleeding in univariate analyses. The absence of significant associations between tumor type, size, or recurrence may reflect insufficient statistical power due to the limited sample size, preventing detection of clinically relevant differences observed in prior studies. Larger prospective studies with adequate sample size calculations are needed to clarify these relationships and validate findings from previous investigations.

Surgical resection of intracranial tumors also carries the risk of post-craniotomy hypertension [16]. Incomplete tumor resection has been linked to a higher incidence of neurological complications, often due to increased intracranial pressure following surgery [20]. Given the hypervascularity of many tumors, complete resection remains the most effective method for achieving durable hemostasis and minimizing bleeding [21,22]. High intraoperative blood loss is a recognized risk factor for POH, likely due to depletion of platelets and coagulation factors. Zetterling et al. reported that a median blood loss of 500 mL significantly increased the likelihood of postoperative hemorrhage, in contrast to other variables such as medication use or pre-existing conditions [11]. Lastly, it is important to acknowledge that no universally accepted definition of POH exists. Indications for reoperation vary between institutions, and minor hemorrhages—although common—often go unmeasured or unreported due to their limited clinical relevance.

The administration of heparin and anticoagulants did not show any significant influence on the risk of postoperative hemorrhage in this study. The heparin dose was prophylactic. It should be emphasized that the number of patients who received heparin or anticoagulants in our study was extremely low. In addition, no consideration was taken of whether or not the patients were receiving antiplatelet medication. Several studies involving diverse patient populations compared various anticoagulation strategies, such as mechanical versus chemical prophylaxis, or chemical prophylaxis versus placebo. These investigations generally suggested that anticoagulants were effective in preventing thromboembolic events, although reported bleeding rates varied considerably across studies [23,24,25,26]. While there is literature on anticoagulation in patients undergoing craniotomy for meningioma, the primary focus has been on whether chemical prophylaxis should be administered. Similarly, prophylactic strategies have been examined in patients undergoing surgery for high-grade glioma [27,28], as well as in those treated for primary malignant brain tumors, where associations between venous thromboembolism and intracranial hemorrhage have also been reported [27]. Comparable findings were also observed in the systematic review of literature conducted by Bianconi et al. The authors’ recommendations suggest that more extensive antithrombotic prophylaxis could potentially improve the prognosis of patients with high-grade glioma [29].

Wang et al. systematically reviewed the risks and benefits of heparin administration in neurosurgical patients [30]. However, available data do not provide clear guidance on the optimal timing or dosing of anticoagulants in patients with prior anticoagulation therapy. Additionally, several studies investigated the incidence and risk factors of postoperative hematoma [11,31,32], though these were limited to hematomas requiring surgical evacuation, which may not reflect all bleeding volumes occurring in or adjacent to the resection cavity. Tumor size was not identified as a relevant risk factor in our analysis. A 2019 retrospective cohort study including intracranial tumor resections found that larger preoperative tumor diameter was associated with a higher risk of postoperative hematoma formation [31]. In the context of large glioma resections, the resulting cavity may promote intradural bleeding due to a greater exposed surface area.

Heparin is frequently used as a bridging therapy during the initiation or interruption of warfarin treatment or in the perioperative setting to prevent thromboembolic complications [23]. This is particularly relevant in neurosurgery, where procoagulant activity may be enhanced by certain tumor types (e.g., meningiomas, gliomas) and reduced fibrinolysis [33]. Some studies indicated that low-dose heparin or low-molecular-weight heparin (LMWH) can be administered safely in neurosurgical patients [34]. However, initiating prophylactic anticoagulation prior to craniotomy has been associated with an increased risk of postoperative hemorrhage. As a result, guidelines recommend maintaining an appropriate interval—at least 18 h—between the last LMWH dose and the surgical procedure [35]. Protamine sulfate can be used intraoperatively to reverse LMWH effects and is titrated according to the severity of bleeding. LMWH remains widely used as a bridging agent during warfarin therapy and for both the prevention and treatment of venous thromboembolism. Nevertheless, preoperative administration is associated with a heightened risk of POH, warranting careful timing of discontinuation [35].

## 5. Conclusions

In this cohort, intraoperative blood loss was the main perioperative factor associated with postoperative hemorrhage after intracranial tumor surgery, which also correlated with longer hospital stays and worse functional outcomes in univariate Analyses. Tumor characteristics showed no significant impact. The retrospective design limits causal inference and increases the risk of selection and information bias. Our single-center setting may not reflect clinical practice across different institutions, thereby limiting the external validity and universality of our findings. Prospective, multicenter studies are warranted to confirm these results in diverse patient populations and clinical contexts. Another possible limitation for the generality of the current results is the missing evaluation of perioperative laboratory parameters in the current analysis. The non-significant association between transfusion and postoperative hemorrhage may be attributable to the small sample size, limiting statistical power to detect clinically relevant differences. The statistical analysis conducted in this study did not reveal any significant association between tumor characteristics or medication use and the occurrence of postoperative bleeding in patients undergoing brain surgery.

## Figures and Tables

**Table 1:** Depicting descriptive statistics and univariate results of homogeneity tests between occurrence and non-occurrence of major hemorrhage during the postoperative period for general tumor characteristics and relevant medication before surgery.

			No Hemorrhage N (%)/ Mean ± SD	Hemorrhage with Operation N (%)/ Mean ± SD	*p*-Value	FDR-Corrected *p*-Values
Tumor characteristics	Recurrence	Yes No	315 (17.37) 1498 (82.63)	4 (12.90) 27 (87.10)	0.637	0.849
	Diagnostic group	Glioma Meningioma Metastases Other	642 (35.06) 414 (22.61) 359 (19.61) 416 (22.72)	10 (32.26) 8 (25.81) 6 (19.35) 7 (22.58)	0.972	1.0
	Side	Left Midline Right	802 (43.99) 255 (13.99) 766 (42.02)	13 (41.94) 3 (9.68) 15 (48.39)	0.749	0.921
	Volume tumor *		44.1 [31.1; 57.0]	45.9 [36.6; 55.3]	0.806	0.921
	Resection volume in %		84.3 ± 27.7	92.3 ± 21.1	0.079	0.253
Medications	NSAID before surgery	Yes No	47 (2.57) 1784 (97.43)	0 (0.00) 31 (100.0)	1.000	1.0
	Heparin before surgery	Yes No	179 (9.78) 1652 (90.22)	5 (16.13) 26 (83.87)	0.224	0.398
	Warfarin before surgery	Yes No	68 (3.71) 1763 (96.29)	2 (6.45) 29 (93.55)	0.326	0.522

* Root-transformation: Indication of the back-transformed means and ranges [mean − SD; mean + SD].

**Table 2:** Depicting the total number of thromboembolic complications during the postoperative patient care period.

		No Hemorrhage N (%)	Hemorrhage with Reoperation N (%)	*p*-Value	FDR-Corrected *p*-Values
Deep venous thrombosis	Yes No	33 (1.80) 1798 (98.20)	1 (3.23) 30 (96.77)	0.438	0.637
Pulmonary artery embolism	Yes No	44 (2.40) 1787 (97.60)	2 (6.45) 29 (93.55)	0.177	0.354

**Table 3:** Depicting surgical procedure-related parameters and their association with postoperative hemorrhage during the postoperative period.

		No Hemorrhage Mean [±SD]	Hemorrhage with Reoperation Mean [±SD]	*p*-Value	FDR-Corrected *p*-Values
Duration of the operation [min]		181.7	210.7	0.111	0.296
Blood loss [mL]		287.8	493.3	0.012	0.064
Duration of stay * (days)		13.9 [13.0; 14.8]	17.7 [16.6; 18.7]	0.016	0.064
Glasgow Outcome Scale (GOS)		4.2 ± 1.0	2.8 ± 1.1	<0.001	<0.001
Karnofsky Performance Scale (KPS)		69.0 ± 18.9	43.2 ± 22.6	<0.001	<0.001
Blood transfusion	Yes No	125 (6.87) 1695 (93.13)	4 (13.33) 26 (86.67)	0.152	0.347

* Root Transformation: Indication of the back-transformed means and ranges [mean − SD; mean + SD].

**Table 4:** Presents the relevant procedure-related parameters in multivariate analysis. The rest of the parameters were non-significant, except for GOS.

	OR	95%; CI	*p*-Value
Blood loss [100 mL]	1.04	0.99; 1.09	0.11
Duration of stay (days)	1.01	0.98; 1.04	0.6
Karnofsky Performance Scale (KPS)	0.99	0.96; 1.02	0.6
Glasgow Outcome Scale (GOS)	0.50	0.26; 0.97	0.040

## Data Availability

The datasets obtained and analyzed during the current study are available from the corresponding author on reasonable request.

## Abbreviations

The following abbreviations are used in this manuscript:

NSAIDs	non-steroidal anti-inflammatory drugs
POH	postoperative hemorrhage
GOS	Glasgow Outcome Scale
KPS	Karnofsky Performance Scale
VTE	venous thromboembolism
LMWH	low-molecular-weight heparin
